# Targeting Weakness With a Combination of Isotonic Exercises in Dermatomyositis With Polyneuropathy: A Case Report

**DOI:** 10.7759/cureus.52873

**Published:** 2024-01-24

**Authors:** Vaishnavi R Waghe, Anam R Sasun, Raghuveer Raghumahanti

**Affiliations:** 1 Department of Neurophysiotherapy, Ravi Nair Physiotherapy College, Datta Meghe Institute of Higher Education and Research, Wardha, IND

**Keywords:** weakness, proprioceptive neuromuscular facilitation, physiotherapy, polyneuropathy, dermatomyositis

## Abstract

Dermatomyositis, an autoimmune inflammatory myositis commonly linked to polymyositis, is marked by inflammatory and degenerative transformations impacting muscles, skin, limb girdles, the neck, and the pharynx. These changes result in symmetrical weakness and diverse levels of muscle atrophy. Uncommonly, the condition may impact the esophagus, lungs, and heart. While dermatomyositis is believed to involve genetic, immunological, and environmental factors, its precise etiology remains elusive. Typically, the classical presentation involves a symmetrical proximal myopathy alongside dermatological manifestations such as a purplish-red rash affecting the face, arms, hands, legs, and other areas. Additional symptoms may include dysphagia, myalgia, fever, and weight loss. The primary objectives of managing dermatomyositis are to address muscular weakness, skin manifestations, and any underlying health concerns. Integral to this management is the utilization of physical therapy and rehabilitation interventions. This study introduces a 23-year-old female patient with a noteworthy medical history covering a duration of two months. The patient reported a chief complaint of persistent thigh pain and a concurrent complaint concerning bilateral weakness in upper and lower extremities. Furthermore, the patient faced the additional challenge of difficulty swallowing. Intriguingly, the patient's clinical presentation was marked not only by the aforementioned symptoms but also by the development of a distinctive facial rash. This facial rash was accompanied by symptoms of stiffness in both small and large joints and a reduction in the range of affected joints. The physiotherapeutic assessment revealed quadriparesis of bilateral upper and lower limbs. The rehabilitation programme for the patient was planned by targeting proprioceptors to increase dynamic trunk balance in patients with DM. The Proprioceptive Neuromuscular Facilitation (PNF) technique employs diagonal movement patterns, thereby proving instrumental in enhancing the patient's daily activities. This methodology serves to optimize the individual's capacity to execute routine daily tasks, promoting independence in their daily life. An investigation like the Nerve Conduction Velocity (NCV) report shows the absence of motor excitation, suggesting motor axonal neuropathy. This approach, comprising isometric, concentric, and eccentric contraction exercises, demonstrated efficacy in mitigating muscular weakness, enhancing motor function, and alleviating the diverse symptoms associated with this condition.

## Introduction

Dermatomyositis (DM), an atypical inflammatory condition, is characterized by distinctive dermatological manifestations and diverse systemic implications. In the initial presentation, patients may display exclusive skin-related symptoms or concurrently experience muscle involvement and extracutaneous manifestations, such as pulmonary complications or an underlying neoplasm [1]. DM, the most prevalent syndrome among idiopathic inflammatory myopathies (IIM), is identified by unique skin manifestations and a spectrum of systemic involvement. The pathophysiological attributes of IIM encompass the existence of autoantibodies and inflammatory exudate within muscles, stemming from a multifaceted interaction between genetic predisposition and environmental factors [2]. Specific environmental conditions, such as UV radiation, certain medications, infectious agents, or lifestyle choices, are believed to contribute to the disease's pathogenesis. It is noteworthy that the diagnostic criteria for IIM are subjects of ongoing discussion, leading to frequent diagnostic challenges [3].

According to the Bohan and Peter criteria, a DM diagnosis is assigned to cases with skin involvement meeting specific criteria: bilateral weakness in the anterior neck flexors and limb-girdle muscles with a gradual onset; elevated serum levels of skeletal muscle enzymes; characteristic electromyographic findings; muscle biopsy demonstrating specific features; and presence of distinctive dermatological features of the DM rash. A diagnosis requires a rash along with muscle symptoms [4].

Clinically, DM typically evolves sub-acutely with the gradual emergence of symmetrical and proximal limb and trunk weakness. Patients may struggle with activities like raising arms, grooming, climbing stairs, or getting up from the ground after falling [5]. While the sensory nervous system and tendon reflexes are usually unaffected, severe cases may require ventilatory support due to diaphragmatic weakness [6]. Cardiovascular involvement, presenting as arrhythmias, myocarditis, and endocarditis during the chronic phase, and issues related to the swallowing apparatus, leading to dysphagia and, in severe cases, aspiration pneumonia, are additional concerns in DM [7-9].

Asymmetrical acute demyelinating inflammatory polyneuropathy is characterized by the World Health Organization primarily as motor neuropathy involving multiple peripheral nerves, often triggered by hypersensitivity reactions to unidentified viruses or allergens [10]. Physical therapy plays a pivotal role in addressing neuromuscular issues, with this study utilizing a combination of isotonic and proprioceptive neuromuscular facilitation (PNF) to enhance functionality [11]. Muscle groups are contracted concentrically, eccentrically, and statically without relaxing [12], and PNF-based exercise regimens prove beneficial in improving patients' functionality [13].

## Case presentation

Patient information

A female patient, age 23, arrived following a two-month history of both upper and lower limb weakness and thigh discomfort. She also had trouble swallowing. The patient had a preceding history of developing a facial rash, stiffness in both small and large joints, and reduced joint mobility, all of which had progressively worsened over the past eight days. Additionally, she experienced facial puffiness for the last two days. In response, comprehensive assessments and investigations were undertaken. Nerve Conduction Velocity (NCV) test shows evidence of motor axonal polyneuropathy, and increased creatine kinase levels were also seen. Both the patient's upper and lower limbs showed symmetric bilateral weakness. The patient received a regimen comprising dextrose normal saline (DNS) infusion, a multiple vitamin injection, pantoprazole 40 mg (Pan 40 injection), emset injection (ondansetron), methotrexate and corticosteroids. The patient reported discomfort during resting at a score of 3/10 and during movement of 8.1/10 on the Visual Analogue Scale (VAS). The discomfort in both the lower and upper limbs was dull and painful, developing gradually and getting worse with movement. Rest and medicine treatment helped to ease it. Interestingly, the reported symptoms did not vary throughout the day.

Clinical findings

After obtaining informed permission, a thorough evaluation of the patient was carried out. The patient's medical history indicates that throughout the previous month, her complaints of discomfort and bilateral weakness in her upper and lower limbs had gotten worse. During the assessment, the patient was placed in a supine reclining posture, and it was noted that her upper and lower limbs' sensory function was intact. However, there was a notable decrease in muscle strength in these areas, indicative of muscular weakness. Muscle power was examined by using the Medical Research Council (MRC) grading system, and the outcomes are provided in Table 1. The observed decrease in muscular strength resulted in constrained joint mobility. Reflexes, encompassing both superficial and deep reflexes, were assessed and revealed an overall reduction in responsiveness. The bilateral plantar reflex exhibited a flexor response, as indicated in Table 2. Muscle tone was gauged using the Tone Grading Scale, with a rating of (+2) ascribed to both the upper and lower extremities. Auscultation identified reduced air entry in the bilateral lower lung zones. Although there was no indication of gastrointestinal or urinary dysfunction, the patient exhibited respiratory challenges and dysphagia. The patient displayed the capacity to sit with moderate assistance yet could not engage in bed mobility exercises or independently perform daily activities, including brushing and eating. Table 3 shows NCV reports.

**Table 1:** Manual Muscle Testing of right and left upper and lower extremity (pre and post-treatment) 2: Complete range of motion (ROM) with the elimination of gravitational forces; 2+: Elimination of gravitational forces or slight resistance, covering less than half the range against gravity; 3-: More than half but less than full ROM against gravity; 3: Complete ROM against gravity; 3+: Full ROM against gravity with slight resistance; 4-: Full ROM against gravity with mild resistance; 4: Full ROM against gravity with moderate resistance

Manual Muscle Testing		Right	Left	Right	Left
Muscles	Variables	Pre-treatment		Post-treatment	
Shoulder	flexors	3-/5	2+/5	3+/5	3/5
	abduction	2/5	2+/5	2+/5	3-/5
Elbow	flexors	2+/5	2+/5	3+/5	3+/5
	extensors	3/5	3-/5	3+/5	4-/5
Wrist	flexors	2+/5	2+/5	3+/5	4-/5
	extensors	2/5	2/5	3+/5	3/5
Hip	flexors	2/5	2/5	3/5	2+/5
	abductors	2/5	2/5	2+/5	3/5
Knee	flexors	2/5	2/5	3+/5	3+/5
Ankle	plantarflexors	3+/5	3+/5	4/5	4/5
	dorsiflexors	2+/5	2+/5	3+/5	3+/5

**Table 2:** Reflexes (pre and post-treatment) 0: absent; +: present but depressed; ++: present/brisk, normal; +++: very brisk/increased; ++++: clonus

Reflexes	Right	Left	Right	Left
	Pre-treatment		Post-treatment	
Biceps	+	+	++	++
Triceps	+	+	++	++
Supinator	+	+	++	++
Abdominal	+	+	++	++
Knee	+	+	++	++
Ankle	+	+	++	++
Plantar	flexor	flexor	flexor	flexor

**Table 3:** NCV report shows absence of motor excitation, suggestive of motor axonal neuropathy NCV: Nerve Conduction Velocity

Test	Stimulation site	Lat., ms	Ampl., mV	Dur., ms	Area, mV×ms	Stim., mA	Stim., ms	Dist., mm	Time, ms	Vel., ms
R, Median										
4	elbow	-	0	-	-	14	0.2	-	-	-
L, Median										
14	elbow	-	0	-	-	48	0.2	-	-	-
R, Ulnar										
15	wrist	-	0	-	-	27	0.2	-	-	-
	elbow	-	0	-	-	30	0.2	-	-	-
L, Ulnar										
16	elbow	-	0	-	-	17	0.2	-	-	-
R, Peroneal										
5	Popliteal fossa	-	0	-	-	38	0.2	-	-	-
L, Peroneal										
10	Head of fibula	-	0	-	-	34	0.2	-	-	-
R, Tibial										
6	Popliteal fossa	-	0	-	-	41	0.2	-	-	-
L, Tibial										
7	Popliteal fossa	-	0	-	-	43	0.2	-	-	-

Test	Site	Lat., ms	Ampl., mV	Dur., ms	Area, mV×ms	Stim., mA	Stim., ms	Dist., mm	Time, ms	Vel., ms
R, Median										
17	Elbow	1.3	18.4	1.2	4.1	9	0.1	140	1.3	108
L, Median										
18	1	1.4	16.6	0.8	3.9	7	0.1	140	1.4	100
R, Ulnar										
19	1	0.8	26.0	0.9	3.8	9	0.1	120	0.75	160
L, Ulnar										
20	Elbow	1.3	20.5	0.9	5.2	4	0.1	120	1.25	96.0

Therapeutic intervention

**Figure 1:**
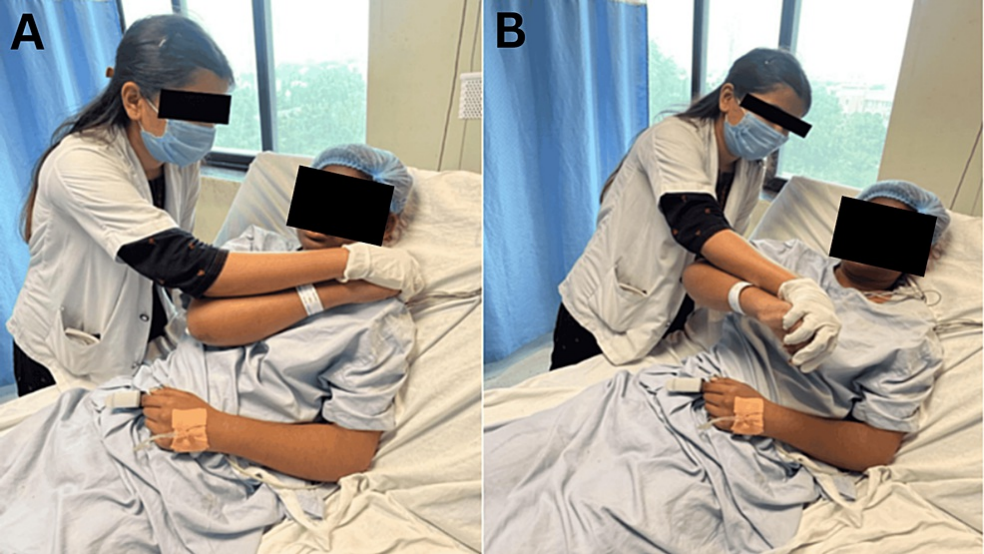
PNF rhythmic initiation in D1 flexion-extension PNF: Proprioceptive Neuromuscular Facilitation A) D1 flexion B) D1 extension

The proprioceptive neuromuscular facilitatory approach was used to regain strength. PNF was performed on trunk, upper and lower limb. The pattern was performed in patients' upper limb D1 and D2 flexion and extension patterns in side-lying and sitting positions. The rest period is vital between the exercise duration to minimize fatigue (Table 4 and Figure 1A, 1B).

**Table 4:** Therapeutic intervention for four weeks PNF: Proprioceptive Neuromuscular Facilitation; D1: Diagonal 1; D2: Diagonal 2

Proprioceptive Neuromuscular Facilitation		
1.	Upper limb	Upper limb PNF exercises encompass various patterns, specifically D1- D2 flexion-extension, flexion–abduction–external rotation and extension–adduction–internal rotation, and flexion–adduction–external rotation and extension–abduction–internal rotation. The prescribed dosage for each exercise involved 10 repetitions per set, with a hold time ranging from 5 to 10 seconds and a relaxation interval of 5 seconds. The progression of the regimen began with rhythmic initiation and advanced to a combination of isotonic movements (concentric, eccentric, isometric), followed by dynamic reversals, stabilizing reversal, rhythmic stabilization, contract-relax, and concluded with the hold-relax technique of PNF. The physiotherapist applied these methods for a duration of 20 minutes each day, five days a week, spanning a period of 6 weeks.
2.	Trunk	Trunk PNF exercises encompass a variety of patterns, including chopping, lifting, bilateral lower extremity flexion with knee flexion for lower trunk flexion and trunk extension, trunk lateral flexion, and right lateral flexion with extension. The prescribed dosage for each exercise involved 10 repetitions per set, with a hold time ranging from 5 to 10 seconds and a relaxation interval of 5 seconds. The progression of the regimen began with rhythmic initiation and advanced to a combination of isotonic movements (concentric, eccentric, isometric), followed by dynamic reversals, stabilizing reversal, rhythmic stabilization, contract-relax, and concluded with the hold-relax technique of PNF. The physiotherapist applied these methods for a duration of 20 minutes each day, five days a week, spanning a period of 6 weeks.
3.	Lower limb	Lower limb PNF exercises involve patterns such as D1- D2 flexion-extension, flexion–abduction–internal rotation and extension–adduction–external rotation, and flexion–adduction–external rotation and extension–abduction–internal rotation. The prescribed dosage for each exercise included 10 repetitions per set, with a hold time of 5 to 10 seconds and a relaxation period of 5 seconds. The progression of the exercises began with rhythmic initiation and advanced to a combination of isotonic movements (concentric, eccentric, isometric), followed by dynamic reversals, stabilizing reversal, rhythmic stabilization, contract-relax, and the hold-relax technique of PNF. The physiotherapist applied these methods for a duration of 20 minutes each day, five days a week, spanning a period of 6 weeks.

Follow-up and outcome measures

Table 5 shows follow-up and outcome measures. A personalized rehabilitation regimen was started, with an emphasis on a combination of isotonic exercises that targeted particular muscle groups. A weekly follow-up evaluation was carried out to track the patient's progress through the rehabilitation regimen. We modified the isotonic exercises according to the patient's progress.

**Table 5:** Outcome measures (pre and post-treatment) VAS: Visual Analogue Scale; WHO-QOL: World Health Organization Quality-of-Life

Outcome Measures	PRE-TREATMENT	POST-TREATMENT
VAS	8/10	3/10
WHO-QOL	50/100	80/100
Barthel Index	20/100	70/100
Dysphagia outcome and severity scale	2/7	6/7
Hughes Scale	4/6	1/6

## Discussion

DM is a complex, chronic inflammatory disorder impacting various organ systems. It is commonly associated with distinctive cutaneous manifestations [14]. Progressive proximal muscle weakening stands out as a common symptom in individuals afflicted with dermatomyositis, impacting a substantial proportion ranging from 53% to 96% of cases [15]. Polyneuropathy, indicating simultaneous dysfunction of multiple peripheral nerves throughout the body, is a recognized aspect of the condition. Healthcare practitioners employ diverse therapeutic modalities to alleviate discomfort and enhance the range of motion in DM patients. These therapeutic approaches include pulsed ultrasound, soft tissue mobilization, stretching exercises, functional strengthening exercises, and cryotherapy. Neuromuscular re-education, utilizing tactile facilitation through slow tapping of muscles, is employed to improve kinesthesia, proprioception, and the development of optimal movement patterns [16].

For patients unable to engage in vigorous physical exercise, percutaneous neuromuscular electrical stimulation (NMES) stimulates skeletal muscle development, enhancing strength and endurance and preventing muscle mass loss. While not universally applicable, clinical trials have shown positive outcomes, particularly with short-term benefits related to skeletal muscle metabolism and muscle mass in critically ill individuals [17]. PNF has demonstrated promising results, including improved patient quality of life, increased strength in lower limb muscles, reduced contractures, and enhanced joint range of motion [18]. PNF represents a stretching technique employed to enhance muscle elasticity, demonstrating efficacy in augmenting both active and passive ranges of motion [19]. Rood's approach, designed explicitly for individuals with motor control challenges, is rooted in physiological principles affirming that sensory stimulation elicits the targeted muscular response. This method is underpinned by four fundamental principles: normalization of muscle tone through sensory stimulation, adherence to ontogenic developmental patterns, incorporation of repetition, and implementation of purposeful movements. According to Rood, sensory stimulation possesses the capacity to either activate or deactivate receptors through facilitation or inhibition, thereby enabling the attainment of the desired muscular response [20].

Active and passive resistive therapeutic exercises serve the dual purpose of enhancing function and mitigating issues such as muscular shortening, contractures, and deformities [21]. A study by Alexanderson revealed that a 12-week home exercise regimen, combining strength training targeting the quadriceps femoris, hamstrings, and abdominal muscles with "careful" stretching exercises, resulted in improved patient function, as assessed using the SF-36 questionnaire, for individuals with both DM and polymyositis [22]. The goal of trunk stabilisation exercises is to improve muscular control that is necessary for bracing the trunk against both internal and external stresses. The rehabilitation programs have prominently centered on the assessment and training of the transversus abdominis muscle, despite the acknowledged contribution of all abdominal muscles to spinal stability. Pelvic proprioceptive neuromuscular stimulation serves to enhance joint proprioception, facilitating improved pelvic control - an essential factor in maintaining overall trunk control and balance. The body can traverse the targeted range of motion through methodologies such as rhythmic initiation, commencing with passive motion and progressing towards active resistance movements [23]. In accordance with the findings of Corrado et al., a statistically significant enhancement in the quality of life was observed in patients with polymyositis and dermatomyositis undergoing physical exercise along with partial blood flow restriction [24]. Concluding the pivotal effect of proprioceptive neuromuscular facilitation in improving dynamic trunk balance in patients with dermatomyositis.

## Conclusions

The presented clinical study highlights the successful application of a comprehensive regimen incorporating isotonic contraction exercises in the management of DM with poly-neuropathy. This approach comprising isometric, concentric, and eccentric exercises, demonstrated efficacy in mitigating muscular weakness, enhancing motor function, and alleviating the diverse symptoms associated with this condition. The favorable patient response to this multidisciplinary strategy underscores the potential for enhanced therapeutic outcomes and improved quality of life among individuals grappling with similar clinical complexities.
